# A Rare Manifestation of Cardiac Myeloid Sarcoma

**DOI:** 10.1016/j.jaccas.2025.106240

**Published:** 2026-01-14

**Authors:** Faaiq N. Aslam, Carlos Vergara-Sanchez, Kevin Tayon, Emily Wolf, James F. Howick V, Pragnesh Parikh, Peter Pollak, Jordan Ray, Charles Ritchie, Prajwal Reddy

**Affiliations:** a Department of Internal Medicine, Mayo Clinic, Jacksonville, Florida, USA; b Department of Cardiovascular Diseases, Mayo Clinic, Jacksonville, Florida, USA; c Department of Hematology and Oncology, Mayo Clinic, Jacksonville, Florida, USA; d Department of Interventional Radiology, Mayo Clinic, Jacksonville, Florida, USA

**Keywords:** AML, cardiac tamponade, case report, myelodysplastic syndrome, myeloid sarcoma, pericardial effusion

## Abstract

**Background:**

Cardiac myeloid sarcoma is a rare hematologic malignancy characterized by myeloid blasts invading cardiac tissue.

**Case Summary:**

A 68-year-old man with myelodysplastic syndrome status post-allogeneic hematopoietic stem cell transplant presented with worsening dyspnea. He was hypoxic but remained hemodynamically stable. Computed tomography angiography chest revealed a large pericardial effusion, and transthoracic echocardiogram confirmed tamponade physiology. The patient underwent an emergent pericardial window, followed by further work-up, including computed tomography, transthoracic echocardiogram, cardiac magnetic resonance, and intracardiac biopsy with novel use of the TLAB device, which identified a right atrial mass consistent with cardiac myeloid sarcoma extending along the interventricular groove with superior vena cava stenosis.

**Discussion:**

Cardiac myeloid sarcoma is a rare primary cardiac malignancy with no established treatment guidelines, but multimodal imaging and accurate tissue diagnosis can help tailor therapy.

**Take-Home Message:**

This case report illustrates a rare case of cardiac myeloid sarcoma and demonstrates feasibility of intracardiac biopsy with novel use of a TLAB device.

## Graphical Abstract

**Figure:**
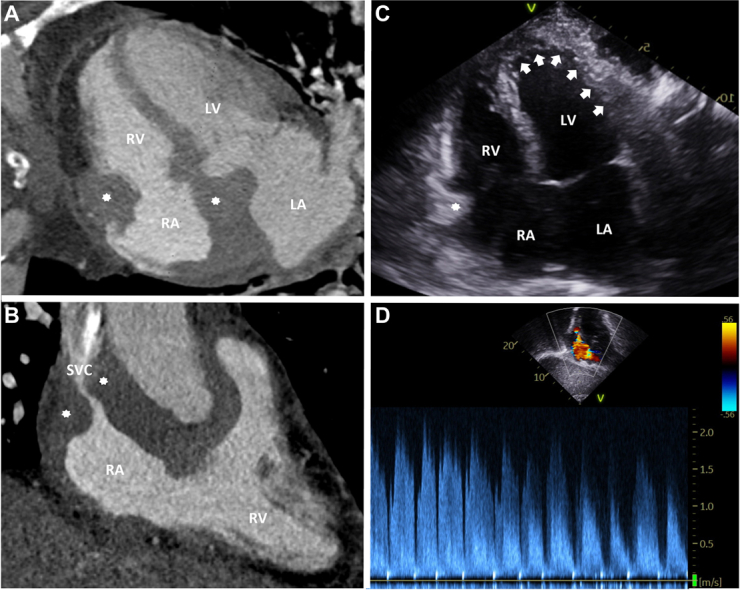

## History of Present Illness

A 68-year-old man presented with 1 to 2 months of progressive exertional dyspnea, which intensified before presentation. On arrival, his blood pressure was 107/74 mm Hg, and his heart rate was 68 beats/min; however, he required supplemental oxygen. Physical examination demonstrated an irregular rhythm with diminished heart sounds, and electrocardiogram demonstrated low voltage atrial fibrillation. Computed tomography angiography of the chest identified a large pericardial effusion, severe superior vena cava (SVC) stenosis, and a mediastinal mass of unclear etiology. A subsequent transthoracic echocardiogram demonstrated a circumferential pericardial effusion with tamponade physiology requiring an emergent pericardial window procedure, which revealed 350 mL of hemorrhagic fluid. His apixaban was subsequently held for 4 weeks after the procedure and then resumed.

**Take-Home Messages**

• Cardiac myeloid sarcoma is an extremely rare, hematologic malignancy characterized by neoplastic myeloid blasts that invade cardiac tissue.
• Diagnosis typically relies on tissue biopsy, with cardiac CT and CMR providing essential information for anatomic localization and characterization, and treatment generally includes low-dose radiation therapy in combination with systemic chemotherapy.
• Core needle biopsy increases diagnostic yield, and intracardiac biopsy with minimally invasive measures is safe and feasible and can help tailor treatment without the need for surgical sampling.

## Past Medical History

This patient had a history of high-risk myelodysplastic syndrome status postmatched unrelated donor allogeneic hematopoietic stem cell transplant (allo-HSCT) 4 years prior, heart failure with reduced ejection fraction secondary to nonischemic cardiomyopathy, and paroxysmal atrial fibrillation on apixaban.

## Differential Diagnosis

The differential diagnosis for the pericardial effusion included acute decompensated heart failure in the setting of his known nonischemic cardiomyopathy, complications related to allo-HSCT such as graft vs host disease, infectious etiologies in the context of his immunosuppression, malignancy including lung cancer, lymphoma, or acute myeloid leukemia, given his history of myelodysplastic syndrome, and idiopathic causes.

## Investigations

**Figure 1:**
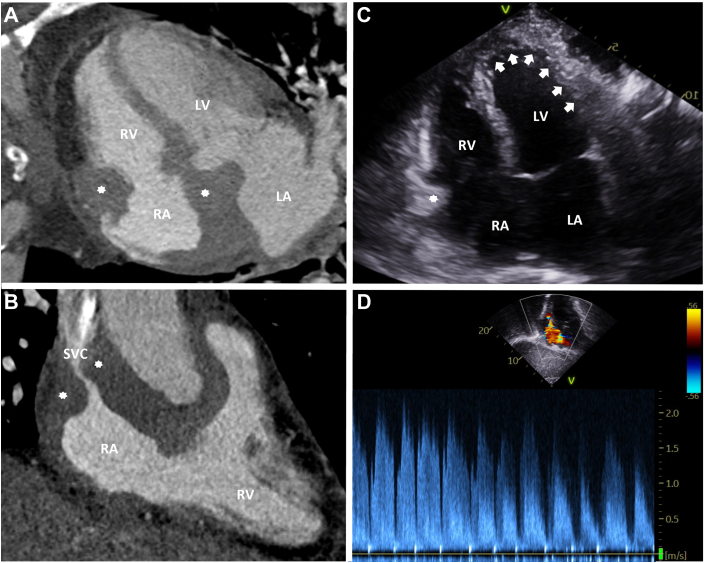
Multimodality Imaging of a Right Atrial Mass With SVC Obstruction (A) Four-chamber multiplanar reconstruction from a cardiac computed tomography angiogram showing a large intracardiac mass (asterisks) involving the RA. (B) Right ventricular inflow-outflow view demonstrating the same mass (asterisks) with SVC stenosis. (C) Transthoracic echocardiogram in the apical 4-chamber view showing the intracardiac mass (asterisk) and a complex pericardial effusion (arrows). (D) Continuous-wave Doppler from the suprasternal view revealing elevated flow velocities in the SVC, consistent with hemodynamically significant SVC obstruction. LA = left atrium; LV = left ventricle; RA = right atrium; RV = right ventricle; SVC = superior vena cava.

Before discharge, a repeat computed tomography scan of the chest was performed to further characterize the mediastinal mass. Imaging demonstrated lobular soft tissue thickening involving the walls of the right atrium (Figure 1A), extending along the interventricular groove, resulting in stenosis of both the SVC and the right coronary artery (Figure 1B). Five days after dismissal from the hospital, a transthoracic echocardiogram (Figures 1C and 1D) showed an ejection fraction of 60%, persistent SVC stenosis, and a probable right atrial mass adherent to the free wall measuring 2.0 × 1.4 cm.

**Figure 2:**
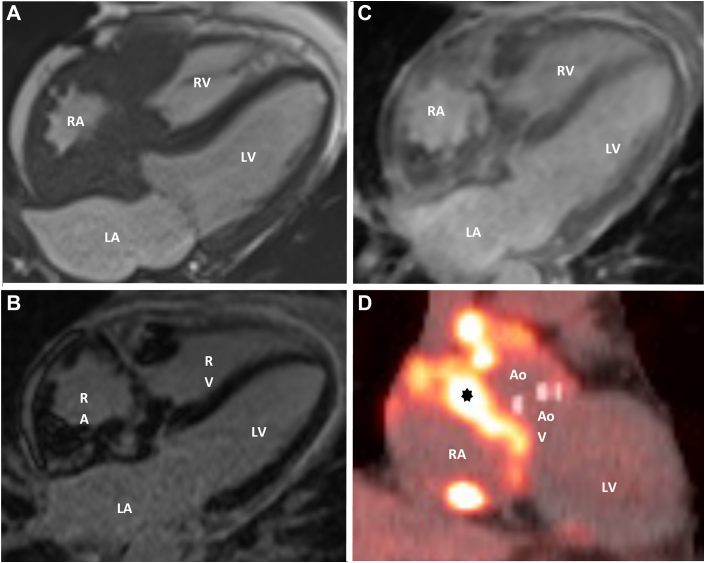
CMR and PET Imaging Demonstrating Infiltrative Right Atrial Mass Consistent With Myeloid Sarcoma (A) Axial cine steady-state free precession cardiac magnetic resonance (CMR) in the 4-chamber view demonstrating an infiltrative mass encasing the RA with extension into the periaortic tissue. (B) Late gadolinium enhancement imaging showing avid enhancement of the right atrial mass with a moderate circumferential pericardial effusion. (C) Delayed postcontrast imaging (600-millisecond inversion time) confirming persistent enhancement of the mass. (D) Cardiac positron emission tomography (PET) revealing a hypermetabolic mediastinal mass (asterisk) encasing the RA and Ao, consistent with progression of previously diagnosed cardiac myeloid sarcoma. Ao = aortic root; AoV = aortic valve; other abbreviations as in Figure 1.

In the interim, the patient developed a left-sided pleural effusion requiring thoracentesis. Cytology analysis of the pleural fluid showed CD34 and CD117 positive cells, raising concern for myeloid sarcoma (MS). Unfortunately, cytology and staining were not performed on pericardial fluid because the cardiac mass had not yet been discovered. A cardiac magnetic resonance (CMR) was subsequently performed (Figures 2A to 2C) and revealed an infiltrative soft tissue mass involving the right atrium, extending from the right ventricular free wall to the right atrioventricular groove, measuring 25.3 mm, with encasement and narrowing of the SVC and, to a lesser extent, the inferior vena cava. CMR also demonstrated restricted right atrial and basal right ventricular motion due to soft tissue encasement of the chambers.

**Figure 3:**
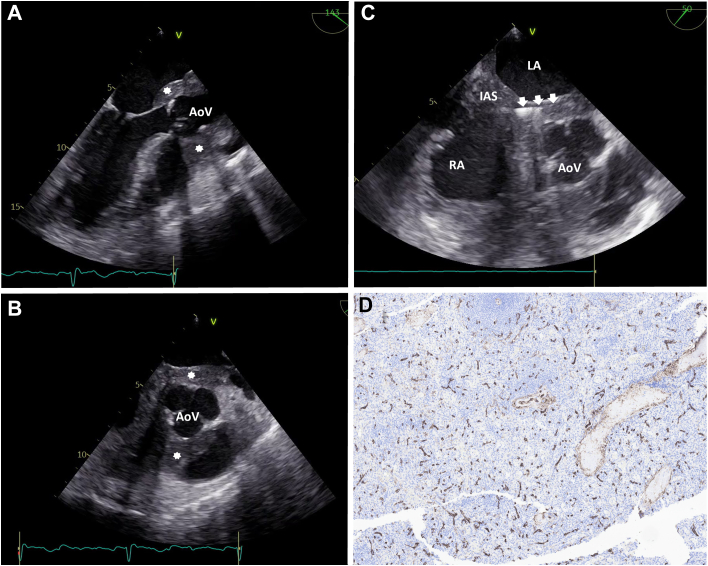
Intra-procedure Transesophageal Echocardiography and Histopathologic Confirmation of Cardiac Myeloid Sarcoma (A, B) Transesophageal echocardiography (TEE) demonstrating a heterogeneous mass (asterisks) encasing the aortic valve (AoV), with infiltration of surrounding structures. (C) TEE-guided transcatheter biopsy of the mass (arrows indicate biopsy needle). (D) Histopathologic specimen from the cardiac biopsy with immunohistochemical staining positive for CD34, consistent with myeloid sarcoma.

Given these findings, biopsies of the right atrial mass and bone marrow were pursued. The right atrial mass was biopsied using the TLAB device (Argon Medical Devices), which was a novel use for this device, because this allows for a core needle biopsy rather than capturing surface tissue that frequently is mostly fibrous tissue or thrombus. The TLAB device was inserted into the femoral vein, and under transesophageal echocardiogram guidance (Figures 3A and 3B, Videos 1 and 2), the directional cannula was steered to the appropriate areas of the cardiac mass and the needle was subsequently advanced to obtain the biopsy (Figure 3C, Video 3). Histopathology of the right atrial mass was positive for CD34 (Figure 3D), C-kit, and myeloperoxidase, confirming the diagnosis of cardiac MS. Next-generation sequencing of the cardiac mass tissue revealed several copy number variations, including *TP53* deletion, *CDKN1B* deletion, *MYC* amplification, and *RAD21* amplification. Interestingly, the bone marrow biopsy showed no evidence of acute myeloid leukemia (AML) but was notable for marked hypocellularity, decreased erythropoiesis, and atypical megakaryocytes. Bone marrow chimerism analysis showed 100% donor and 0% recipient DNA. Next-generation bone marrow sequencing showed no pathogenic alterations, and chromosome analysis showed a normal male karyotype, indicating no apparent clonal abnormality. Thus, the diagnostic work-up confirmed isolated, extramedullary disease.

## Management

To treat the patient's cardiac MS, low-dose radiation therapy was initiated (15 Gy in 10 fractions). After completion, he was started on systemic treatment with venetoclax and a hypomethylating agent (initially azacitidine, and later transitioned to decitabine). Computed tomography angiography chest showed decreased size of the infiltrative cardiac mass and a decreased left pleural effusion. However, 5 months later, a positron emission tomography scan showed disease progression (Figure 2D). As a result, he was subsequently started on combination chemotherapy with cladribine (purine antimetabolite) and cytarabine (DNA polymerase inhibitor) alongside venetoclax.

## Outcome and Follow-Up

Despite treatment, the patient required multiple hospitalizations for complications related to both his malignancy and its treatment, including aspiration pneumonia and hypoxic respiratory failure from recurrent pleural effusions. Follow-up positron emission tomography imaging demonstrated continued disease progression despite cladribine, cytarabine, and venetoclax treatment. Cytologic analysis of pleural fluid ultimately confirmed transformation to AML. Due to his persistent respiratory failure unresponsive to aggressive medical management, the patient transitioned to hospice care and died peacefully.

## Discussion

In this report, we describe a case of MS, a tumor consisting of myeloid blasts occurring at an anatomic site other than the bone marrow.^1^^,^^2^ It most commonly occurs with an already present bone marrow neoplasm, most commonly AML.^1^^,^^2^ However, cases of de novo MS without any underlying bone marrow malignancy have also been described and, if left untreated, may progress to AML.^1^ Although it can occur at any anatomic site, it frequently involves the skin/soft tissue and lymph nodes, with extremely rare isolated cardiac infiltration.^1^^,^^3^ Among patients with cardiac involvement, <1% develop clinical symptoms.^3^

Current knowledge of cardiac MS is limited, given most literature consists of case reports. In a review by Gautam et al,^4^ only 30 reported cases of cardiac MS have been identified between 1960 and 2016.^3^, ^4^ Of these, 6 cases had no evidence of bone marrow involvement, and 4 developed cardiac MS after allo-HSCT.^3^^,^^4^ The clinical presentations were heterogeneous and included pericarditis, tachycardia, palpitations, dyspnea, arrhythmias, and heart block.^4^

Our case is unique in that the patient developed cardiac MS in the absence of bone marrow involvement and after allo-HSCT—a presentation that has only been reported 4 times prior. One such case, described by Tirado et al,^5^ involved a 30-year-old man with acute promyelocytic leukemia status post-allogenic bone marrow transplant who developed multifocal MS, including the right atrium. Cytogenic and fluorescence in situ hybridization studies of the atrial mass identified fusions on chromosomes 7 and 15, which were hypothesized to contribute to the disease's atypical presentation.^5^ Next-generation sequencing revealed several genetic alterations of our patient's cardiac MS, including *TP53* deletion, *CDKN1B* deletion, *MYC* amplification, and *RAD21* amplification. *TP53* mutations are more frequently observed in therapy-related AML and myelodysplasia-related rather than de novo AML.^6^ *TP53*-mutated AML is associated with a poor response to conventional treatment and dismal outcomes.^6^ It is unclear whether *TP53* mutation is linked with cardiac MS, but it may have contributed to the refractory nature of our patient's disease.

Clinical presentations of cardiac MS can be variable and often depend on the location and extent of cardiac involvement. In our case, the patient presented primarily with pericardial effusion and cardiac tamponade. Pericardial involvement in MS has been previously reported. Tsai et al^7^ described a case of a 16-month-old girl with a mediastinal MS with pericardial invasion who also presented with pericardial effusion requiring intervention. That patient later developed worsening SVC syndrome due to progressive compression and ultimately died from sepsis related to chemotherapy-induced neutropenia.^7^ In contrast, our patient had right atrial involvement rather than mediastinal disease. Although imaging demonstrated SVC stenosis, he did not develop overt SVC syndrome.

Currently, there are no established guidelines for the management of cardiac MS due to its rarity. Diagnosis requires a high index of clinical suspicion because presenting signs and symptoms often overlap with more common cardiac conditions.^4^ Histologic confirmation is the gold standard for diagnosis. However, modern imaging modalities like cardiac computed tomography and CMR play an essential role in the characterization of cardiac masses and in guiding diagnostic and therapeutic planning.^4^^,^^8^ For example, volume-perfusion cardiac computed tomography scans can assess tissue vascularity, whereas electrocardiogram-gated CMR offers enhanced functional and structural assessment.^4^^,^^8^

In addition to the lack of diagnostic guidelines, there are also no standardized treatment regimens for cardiac MS. Given the potential for cardiac compromise, treatment strategies must balance therapeutic efficacy with minimization of cardiotoxicity.^4^ Although anthracyclines are commonly used to treat AML, their known cardiotoxic effects often preclude their use in patients with cardiac involvement.^4^ As a result, alternative regimens with reduced cardiac risk are critical in this population. Yang et al^9^ reported a case of a 19-year-old with cardiac MS who achieved complete remission with a combination of the hypomethylating agent azacytidine followed by targeted radiation therapy (24 Gy in 12 fractions).^4^, ^9^ In a similar approach, our patient was treated with radiation (15 Gy in 10 fractions) followed by systemic therapy consisting of a hypomethylating agent in combination with venetoclax.^10^

Cardiac MS is an exceedingly rare condition with no established management guidelines. Its clinical presentation is often nonspecific and may mimic more common cardiac conditions, necessitating a high index of suspicion in patients, particularly in patients with hematologic malignancies or a history of stem cell transplantation. Although tissue biopsy remains the gold standard for diagnosis, cardiac computed tomography and CMR are increasingly essential to characterize these masses. Moreover, the use of low-dose targeted radiation combined with systemic chemotherapy that minimizes cardiotoxicity represents a pragmatic and potentially effective therapeutic approach.

## Funding Support and Author Disclosures

The authors have reported that they have no relationships relevant to the contents of this paper to disclose.

## Appendix

Video 1 Mid-esophageal long-axis view on intra-procedure transesophageal echocardiogram (TEE) demonstrating heterogenous mass encasing the aortic valve (AoV).

Video 2 Mid-esophageal aortic valve short-axis view on intra-procedure transesophageal echocardiogram (TEE) demonstrating heterogenous mass encasing the aortic valve (AoV).

Video 3 Mid-esophageal right ventricular inflow outflow view on intra-procedure transesophageal echocardiogram (TEE) guided transcatheter biopsy of the mass.

## References

[1] Ramia de Cap M., Chen W. (2023). Myeloid sarcoma: an overview. Semin Diagn Pathol.

[2] Almond L.M., Charalampakis M., Ford S.J., Gourevitch D., Desai A. (2017). Myeloid sarcoma: presentation, diagnosis, and treatment. Clin Lymphoma Myeloma Leuk.

[3] Salisbury T., Al-Mohammad A., Pirzada O. (2023). Isolated cardiac involvement of a primary myeloid sarcoma: a case report of an unusual cause of pulmonary oedema. Eur Heart J Case Rep.

[4] Gautam A., Jalali G.K., Sahu K.K., Deo P., Ailawadhi S. (2017). Cardiac myeloid sarcoma: review of literature. J Clin Diagn Res.

[5] Tirado C.A., Chen W., Valdez F. (2010). Unusual presentation of myeloid sarcoma in a case of acute promyelocytic leukemia with a cryptic PML-RARA rearrangement involving multiple sites including the atrium. Cancer Genet Cytogenet.

[6] Shahzad M., Amin M.K., Daver N.G. (2024). What have we learned about TP53-mutated acute myeloid leukemia?. Blood Cancer J.

[7] Tsai M.H., Yang C.P., Chung H.T., Shih L.Y. (2009). Acute myeloid leukemia in a young girl presenting with mediastinal granulocytic sarcoma invading pericardium and causing superior vena cava syndrome. J Pediatr Hematol Oncol.

[8] Dörfel D., Häntschel M., Federmann B. (2016). Cardiac myeloid sarcoma: multimodality radiologic imaging features and pathologic correlation. Am J Med.

[9] Yang W.C., Yao M., Chen Y.H., Kuo S.H. (2016). Complete response of myeloid sarcoma with cardiac involvement to radiotherapy. J Thorac Dis.

[10] DiNardo C.D., Jonas B.A., Pullarkat V. (2020). Azacitidine and venetoclax in previously untreated acute Myeloid leukemia. N Engl J Med.

